# Intranasal insulin improves mitochondrial function and attenuates motor deficits in a rat 6‐OHDA model of Parkinson's disease

**DOI:** 10.1111/cns.13609

**Published:** 2021-01-26

**Authors:** Farideh Iravanpour, Leila Dargahi, Mohsen Rezaei, Masoud Haghani, Reza Heidari, Neda Valian, Abolhassan Ahmadiani

**Affiliations:** ^1^ Neuroscience Research Center Shahid Beheshti University of Medical Sciences Tehran Iran; ^2^ Neurobiology Research Center Shahid Beheshti University of Medical Sciences Tehran Iran; ^3^ Department of Toxicology Faculty of Medical Sciences Tarbiat Modares University Tehran Iran; ^4^ Histomorphometry and Stereology Research Center Shiraz University of Medical Sciences Shiraz Iran; ^5^ Pharmaceutical Sciences Research Center Shiraz University of Medical Sciences Shiraz Iran

**Keywords:** 6‐OHDA, Drp‐1, insulin, mitochondrial dysfunction, Parkinson's disease

## Abstract

**Aims:**

Experimental and clinical evidences demonstrate that common dysregulated pathways are involved in Parkinson’s disease (PD) and type 2 diabetes. Recently, insulin treatment through intranasal (IN) approach has gained attention in PD, although the underlying mechanism of its potential therapeutic effects is still unclear. In this study, we investigated the effects of insulin treatment in a rat model of PD with emphasis on mitochondrial function indices in striatum.

**Methods:**

Rats were treated with a daily low dose (4IU/day) of IN insulin, starting 72 h after 6‐OHDA‐induced lesion and continued for 14 days. Motor performance, dopaminergic cell survival, mitochondrial dehydrogenases activity, mitochondrial swelling, mitochondria permeability transition pore (mPTP), mitochondrial membrane potential (Δψ_m_), reactive oxygen species (ROS) formation, and glutathione (GSH) content in mitochondria, mitochondrial adenosine triphosphate (ATP), and the gene expression of PGC‐1α, TFAM, Drp‐1, GFAP, and Iba‐1 were assessed.

**Results:**

Intranasal insulin significantly reduces 6‐OHDA‐induced motor dysfunction and dopaminergic cell death. In parallel, it improves mitochondrial function indices and modulates mitochondria biogenesis and fission as well as activation of astrocytes and microglia.

**Conclusion:**

Considering the prominent role of mitochondrial dysfunction in PD pathology, IN insulin as a disease‐modifying therapy for PD should be considered for extensive research.

## Graphical abstract

Intranasal administration of insulin causes direct delivery of insulin from nose to the various areas of the brain including substantia nigra and striatum. We showed that insulin can improve mitochondrial function indices and modulate mitochondrial biogenesis and fission as well as astrocytes and microglia activity which overall leads to improved motor performance in a rat model of Parkinson's disease.

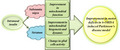

## 1 INTRODUCTION

Parkinson's disease (PD) is the second common neurodegenerative disorder associated with progressive loss of dopaminergic (DA) neurons in the substantia nigra pars compacta (SNpc) and degeneration of projecting dopaminergic terminals to the striatum, which is linked to motor deficits.^1^ PD has a multifactorial pathogenesis, involving both genetic and environmental factors.^1^, ^2^ The most important mechanisms implicated in development of PD include aggregation of misfolded proteins, mitochondrial dysfunction, energy failure, oxidative stress, and dysregulated calcium homeostasis.^3^ Current research extensively focuses on finding new early diagnostic approaches and introducing molecular targets for disease‐modifying treatments specified for underlying causes of PD and reducing DA neuronal loss.^4^, ^5^, ^6^

Regarding the neuroprotective effects of insulin in the brain,^7^ and the probable causative role of insulin resistance in PD pathogenesis,^8^ emerging studies continue to demonstrate the beneficial effects of intranasal (IN) insulin in both animal models of PD and humans in clinical trials.^9^, ^10^, ^11^ The insulin receptors are widely found in basal ganglia and SN and insulin is described as a critical regulator of energy balance, neuronal survival, and growth, neurotransmission, and maintenance of synapses in brain.^12^ However, little is known about the involving mechanisms in neuroprotective effects of insulin in PD.

Nigrostriatal neurons have a high energy requirement and dysfunction of mitochondria, as the source of energy supply, plays a key role in the development of PD.^13^, ^14^ Mitochondrial dysfunction manifested by reduced adenosine triphosphate (ATP) production and calcium buffering capacity, as well as impaired degradation of damaged mitochondria through mitophagy and replacement with new functional mitochondria through biogenesis, has been linked with PD.^15^ Mitochondrial Ca^+2^ overloading activates the opening of mitochondrial membrane transition pore (mPTP) and consequently causes the collapse of mitochondrial membrane potential (Δψ_m_), reduced ATP level, release of pro‐apoptotic mediators into the cytosol and finally mitochondria‐mediated cell death.^14^ Furthermore, mitochondrial dynamic (fission and fusion) and biogenesis have essential roles in neuronal survival.^14^, ^16^ The main factor involved in mitochondrial fission is dynamin‐related protein 1 (Drp‐1), a dynamin‐like GTPase, which is recruited to the fission sites of outer membrane of mitochondria.^16^ Production of new mitochondria is principally regulated by peroxisome proliferator‐activated receptor gamma (PPARγ) coactivator 1α (PGC‐1α) which activates the expression of transcription factors like nuclear respiratory factors (NRF1 and 2) and mitochondrial genes encoded by nucleus such as mitochondrial transcription factor A (TFAM).^17^ Emerging evidence suggests mitochondria as a novel and highly relevant therapeutic target to reduce neurodegeneration in PD.^18^

Inadequate insulin signaling or impaired cellular response to insulin, termed as insulin resistance, is among the causes of mitochondrial dysfunction in the development and progression of PD.^19^ Insulin act as a modulator of electron transport chain activity and the main regulator of mitochondrial biogenesis, through activation of the PI3K/Akt pathway.^20^ In this study, we investigated the effects of IN insulin administration on various indices of mitochondrial function in the striatum of 6‐OHDA PD modeled rats. In parallel, mitochondrial biogenesis and mitochondrial fission, behavioral motor performance, DA neurons survival, and glial cells activation markers were also assessed.

## 2 MATERIALS AND METHODS

### 2.1 Chemicals

Desipramine‐HCl and regular human insulin were purchased from Exir Co. (Tehran, Iran). 6‐hydroxydopamine hydrochloride (6‐OHDA), apomorphine, paraformaldehyde (PFA), hydrogen peroxide, trichloroacetic acids (TCA), hydroxymethyl aminomethane hydrochloride (Tris‐HCl), and Rhodamine 123 were purchased from Merck Co. (Darmstadt, Germany). Horseradish peroxidase (HRP), 3, 3′‐Diamino benzidine tetrahydrochloride (DAB), mannitol, sucrose, 4,2‐hydroxyethyl,1‐piperazineethanesulfonic acid (HEPES), 3‐[4,5dimethylthiazol‐2‐yl]‐2,5‐diphenyltetrazolium bromide (MTT), dimethyl sulfoxide (DMSO), fatty acid‐free bovine serum albumin (BSA), dithiobis‐2‐nitrobenzoic acid (DTNB), glutathione (GSH), 2′,7′‐dichlorofluorescein diacetate (DCFH‐DA), potassium chloride (KCl), ethylene di‐amine tetra acetic acid (EDTA), ethylene glycol‐bis (2‐aminoethyl ether)‐N,N,N′,N′‐tetra acetic acid (EGTA) and Bradford reagent were purchased from Sigma Chemical Co. (St. Louis, MO, USA). Rabbit polyclonal anti‐TH antibody was purchased from Abcam Co. (USA). SYBR Green Real‐Time PCR Master Mix was purchased from Ampliqon Co (Denmark).

### 2.2 Animals

Male Wistar rats (300–325 g) from the breeding colony of Neuroscience Research Center were housed as five in a cage under a 12 h‐light/12 h‐dark cycle. Food and water were available ad libitum, and the temperature of room was set at 23 ± 2ºC. The rats were allowed 5–6 days of habituation to the animal colony. The experiments were accomplished under the National Institutes of Health guide for the care and use of laboratory animals and approved by the ethics committee for animal research of the Shahid Beheshti University of Medical Sciences (IR.SBMU.MSP.REC.1397.54).

### 2.3 Stereotaxic surgery

One hour before the surgery, desipramine‐HCl (25 mg/kg) was injected intraperitoneal (i.p.) to prevent uptake of 6‐OHDA into noradrenergic terminals.^21^ Then the rats were anesthetized with ketamine/xylazine (80/20 mg/kg, i.p.) and were placed on a stereotaxic frame with a rat adaptor. A total of 20 μg 6‐OHDA (in 4 μl normal saline with 0.2 mg/ml ascorbic acid) was injected into the right medial forebrain bundle (MFB) (AP: 4.3, ML: 1.6, DV: 8.2)^22^ using Hamilton syringe during 4 min. After an additional 5 min, the needle was gently pulled out. The control rats received the same volume of vehicle (normal saline with 0.2 mg/ml ascorbic acid).

### 2.4 Insulin administration and experimental groups

From day three after surgery, rats received regular human insulin (4IU/day; 2IU in each nostril, for 14 days) or normal saline intranasally in an upright head position, as previously described.^23^ There were four experimental groups including; 1) Sham: received vehicle injection in MFB, 2) 6‐OHDA: received 6‐OHDA in MFB, 3) 6‐OHDA+Ins: received 6‐OHDA in MFB, and intranasal insulin, and 4) 6‐OHDA+Sal: received 6‐OHDA in MFB, and intranasal normal saline. Blood glucose levels were measured by tail sampling, 30 min after each intranasal administration, using a Contour Blood Glucose Monitoring System and the body weight of animals was recorded daily (*n* = 6/group). The day after the last intranasal treatments (day 17 post‐surgery) rats were subjected to behavioral tests (*n* = 7–10/group) and on day 18 post‐surgery were sacrificed for immunohistochemical staining (*n* = 3/group), mitochondrial assessments (*n* = 6/group), and qPCR analysis (*n* = 3–4/group).

### 2.5 Behavioral tests

All rats were trained on the behavioral tests (narrow beam and rotarod tests) for two consecutive days before stereotaxic surgery. One day after the last insulin/normal saline administration, the rats were subjected to beam, rotarod and apomorphine‐induced rotational tests.

#### 2.5.1 Apomorphine‐induced rotation

The rats were placed in the chamber to habituation. After 30 min, they received 0.25 mg/kg apomorphine (dissolved in a 0.2 mg/ml ascorbic acid in normal saline) subcutaneously (s.c.). Ipsilateral and contralateral rotations were recorded by a fixed camera for 20 min. The results are expressed as net rotations (contralateral turns ‐ ipsilateral turns).^11^

#### 2.5.2 Rotarod test

Motor performance was evaluated using a rotarod apparatus as previously described.^24^ The rats were trained five times a day for two consecutive days, before stereotaxic surgery, until they could stay on the rotating rod for 300 s. In the first training day, the rotarod speed was constant at 10 rpm, and in the second day, it was accelerating from 5 to 20 rpm during 300 s. In the test sessions, the speed was increased from 5 to 40 rpm over 300 s, and each rat performed five trials with 300 s cutoff. The average of five trials was considered as the final score.

#### 2.5.3 Narrow beam test

Narrow beam apparatus is a long wooden beam (100 cm in length, 4 cm wide and 3 cm thick) elevated 80 cm above the ground. A line is drawn 20 cm from the start end of beam, and a cage is placed at the other end. During the training and testing sessions, the rats were placed entirely within the 20 cm starting zone facing its home cage, and the total time of walking on the beam to reach the home cage was recorded. Before the surgery, the animals received two consecutive days of training each consisting of five trials. Each rat was subjected to five trials with 120 s cutoff in testing sessions, and the average of five trials was considered as the final score.^25^

### 2.6 Immunohistochemistry

The rats were anaesthetized with ketamine/xylazine (80/20 mg/kg) and transcardially perfused with normal saline, followed by 4% PFA. The brains’ right hemisphere (ipsilateral to lesion) were removed, and kept in the same fixative for 24 h, and in 30% sucrose for 72 h. Afterward, each brain is placed on to a pre‐labeled tissue base mold, embedded with optimal cutting temperature compound (OCT) and stored at −80°C. Coronal sections (10 μm) were cut on a cryostat and used for immunostaining. In brief, frozen sections were left at room temperature for 10–20 min. Then they were put in methanol for 30 s and washed in distilled water for few seconds. Endogenous peroxidase was inactivated by 3% aqueous solution of hydrogen peroxide, and 10% normal goat serum (30 min) was used to block non‐specific binding sites. The sections were incubated with rabbit polyclonal anti‐TH antibody (1:1000) over night at 4°C. TH‐immunoreactivity was detected by HRP conjugated secondary antibody and visualized using liquid DAB followed by counterstaining with Mayer's hematoxylin. The boundary between SNpc and ventral tegmental area (VTA) was determined at ×40 magnification, and the immunoreactive neurons were counted at ×400 magnification. Three animals were used in each group, and the values from at least three sections were averaged for each animal.

### 2.7 Extraction of striatal mitochondria

The mitochondria were isolated from right striatum (ipsilateral to lesion) as previously described.^26^, ^27^ Briefly, the animals were anesthetized using ketamine/xylazine (80/20 mg/kg, i.p.) and the striatum was rapidly dissected from the brain on ice and washed with cold normal saline. The tissues were homogenized in a buffer containing 70 mM mannitol, 220 mM sucrose, 0.5 mM EGTA, 2 mM HEPES, 0.1% essentially fatty acid‐free BSA (pH = 7.4) at a 10:1 ratio of isolation buffer to striatum tissue (v:w). Then, the homogenate was centrifuged at 600*g* (10 min at 4°C) to remove cells debris. The supernatants were additionally centrifuged at 10,000*g* (10 min at 4°C) to precipitate the mitochondria. This step was repeated at least three times by fresh isolation buffer. The obtained mitochondrial fraction was resuspended into the same buffer as above but without BSA, at a final concentration of 20mg protein/ml, determined by the Bradford reagent.

### 2.8 Mitochondrial evaluation

#### 2.8.1 Mitochondrial dehydrogenases activity

3‐[4,5dimethylthiazol‐2‐yl]‐2,5‐diphenyltetrazolium bromide assay was used to examine the mitochondrial dehydrogenases activity in isolated mitochondria.^27^ In brief, mitochondrial suspension (1 mg protein/ml) was incubated with 0.4% of MTT (37°C, 30 min in the dark) in a buffer containing 1 mM EDTA, 320 mM sucrose, and 10 mM Tris‐HCl (pH = 7.4). Samples were centrifuged (10,000*g*, 15 min) and the purple formazan crystals were dissolved in 1 ml DMSO. The optical density of the dissolved formazan (100 μl in 96‐well plate) was measured at 570 nm using a FLUOstar Omega^®^ multifunctional microplate reader.

#### 2.8.2 Mitochondrial swelling and permeabilization

Mitochondrial swelling was evaluated by the light scattering method as described before.^26^, ^27^ Briefly, the isolated mitochondria (0.5 mg protein/ml) were suspended in swelling buffer (125 mM sucrose, 65 mM KCl, 10 mM HEPS‐KOH, 10 mM CaCl_2_, pH = 7.2). The light absorbance was monitored at two time points during 30 min (at 540nm and constant temperature 25°C) with the multifunctional microplate reader. A decrease in light absorbance is related to increased mitochondrial swelling and permeabilization. The difference between the absorbance of two time points was reported as maximal mitochondrial swelling amplitude (ΔOD at 540 nm).

#### 2.8.3 Mitochondrial membrane potential

The uptake of the cationic fluorescent probe, Rhodamine 123, was used to estimate of mitochondrial depolarization.^26^, ^27^ The isolated mitochondria (0.25 mg protein/ml) were incubated with 0.2 μM Rhodamine 123, in a buffer containing 125 mM sucrose, 10 mM HEPES, 65 mM KCl, and pH = 7.2 (20 min at 37°C in the dark). The samples then were centrifuged (10,000*g*, 5 min at 4 °C), and their fluorescence intensity was evaluated using the multifunctional microplate reader at the excitation and emission wavelengths of 485 nm and 525 nm, respectively.

#### 2.8.4 Reactive oxygen species (ROS) formation

The fluorescent probe DCFH‐DA was used to evaluate ROS formation in mitochondria.^26^, ^27^ Extracted mitochondria (0.25 mg protein/ml) were resuspended in the respiratory buffer (125 mM sucrose, 5 mM sodium succinate, 65 mM KCl, 10 mM HEPES, pH = 7.2) with 10 μM DCFH‐DA. Finally, the fluorescence intensity of DCFH‐DA was measured at the excitation and emission wavelengths, 485 nm and 525 nm respectively, using the multifunctional microplate reader.

#### 2.8.5 Mitochondrial GSH content

Mitochondrial GSH level was determined using DTNB, as an indicator of GSH, by a spectrophotometric method.^27^ The mitochondrial suspension (0.5 mg protein/ml) was treated with TCA to a final concentration of 10% (w/v), and centrifuged at 15,000*g* (1 min at 4ºC) to remove denatured proteins. Afterward, 100 μl of DTNB (0.04% in phosphate buffer) was added, and intensity of produced yellow color was measured at 412 nm using the multifunctional microplate reader.

#### 2.8.6 Mitochondrial ATP content

The ATP level of mitochondria was measured using a luciferase‐luciferin based kit (ENLITEN^®^ from Promega, Madison, WI).^28^ The samples and buffer solutions were prepared according to the kit instructions. Briefly, isolated mitochondria (1 mg protein/ml) were treated with 100 μl TCA (0.5% w: v) and centrifuged (10,000*g*, 10 min, 4°C). Then, 100 μl of the supernatant was mixed with 100 μl of ATP kit content. The luminescence intensity was detected at 560 nm using the multifunctional microplate reader.

### 2.9 RNA isolation and qPCR Protocol

The rats were decapitated and right striatum (ipsilateral to lesion) was immediately dissected on ice, and total RNA was extracted using RNA extraction kit (Cinnagen Inc., Iran) according to the manufacturer’s instructions. The quality and purity of extracted RNA were respectively evaluated by electrophoresis visualization of 28S and 18S ribosomal RNA bands and the spectrophotometric A260/A280 ratio using Nanodrop^TM^ spectrophotometer (Nanodrop; Thermo Fisher Scientific, Wilmington, DE, USA), and then, the extracted RNA stored at −80°C until cDNA synthesis. The cDNA was synthesized using 1μg RNA according to the manufacturer's protocols RevertAid™ First Strand cDNA Synthesis kit (Fermentas Inc., Germany). The cDNA was used to quantitatively measure the expression of target genes using SYBR Green Real‐Time PCR Master Mix reagents on ABI System (USA). The cycling conditions were as follows: activation 5min 95°C, denaturation 30 s 95°C, annealing 30 s, and extension 30 s 72°C. The relative genes expression was calculated using 2^‐ΔΔCt^ formula. Primers sequences used for qPCR are shown in Table 1.

**TABLE 1:** Primer sequences used for qPCR

Gene	Forward primer (5′ 3′)	Reverse primer (5′ 3′)
GFAP	AACCGCATCACCATTCCTGT	TCCTTAATGACCTCGCCATCC
Iba1	TCGTCATCTCCCCACCTAAG	ATCAAACTCCATGTACTTCGTCTTG
PGC1α	GTGCAGCCAAGACTCTGTATGG	GTCCAGGTCATTCACATCAAGTTC
TFAM	AGAGTTGTCATTGGGATTGG	CATTCAGTGGGCAGAAGTC
Drp1	ACAACAGGAGAAGAAAATGGAGT	ATCCACAAGCGTCAGGTTGA
β2M	CGTGCTTGCCATTCAGAAA	ATATACATCGGTCTCGGTGG

### 2.10 Statistical analysis

Statistical analyses were performed using 6th version of GraghPad Prism. Data are expressed as mean ± standard error of mean (SEM). Kolmogorov‐Smirnov (KS) normality test indicated that the data have normal distribution; therefore, comparisons were done using parametric tests, two‐way analysis of variance (ANOVA) with repeated measures and one‐way ANOVA followed by Tukey's post hoc. Statistical significance was set at *p* < 0.05.

## 3 RESULT

### 3.1 Intranasal insulin administration had no effect on the body weight and blood glucose levels

**FIGURE 1:**
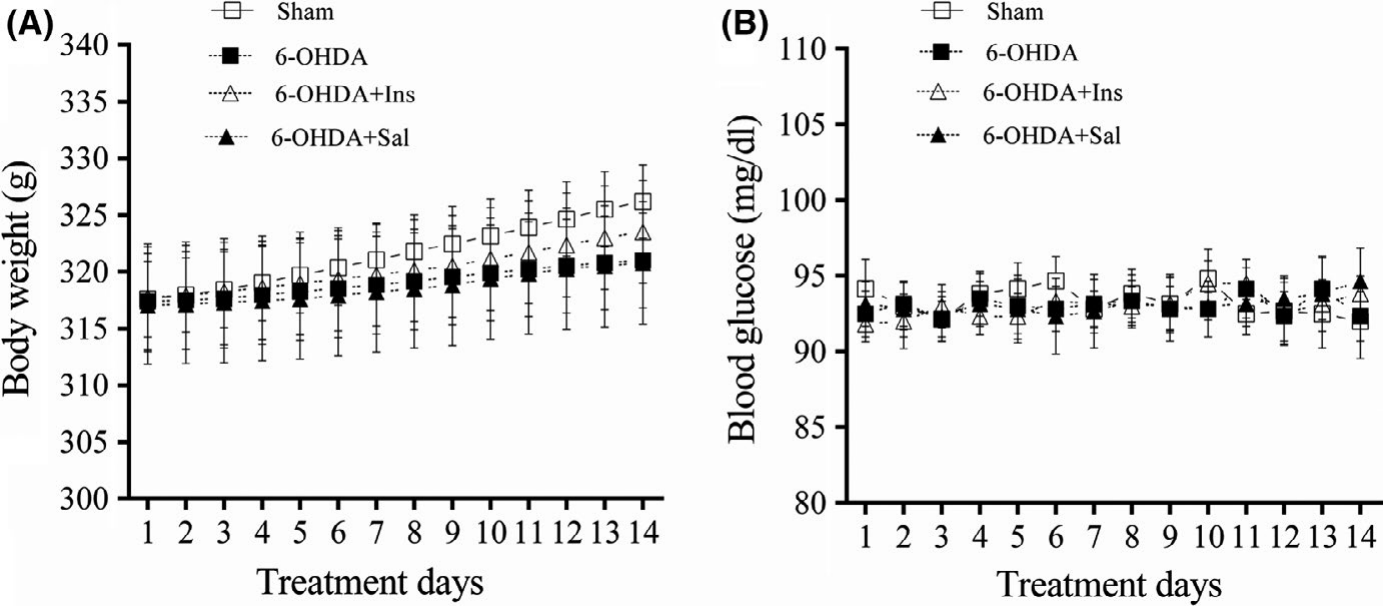
Changes in the body weight and blood glucose levels. The treatments were started from day three after stereotaxic surgery and 6‐OHDA injection and continued for 14 days. The (A) body weight and (B) blood glucose levels were not different between the experimental groups. Values are expressed as means ± SEM (*n *= 6).

Two‐way ANOVA with repeated measures indicated no significant effect of treatment [F _(3, 20)_ = 0.531, *p* > 0.05] on the body weight. However, there were a significant effect of time × treatment interaction [F _(39, 260)_ = 46.63, *p* < 0.001] and time [F _(13, 260)_ = 1,116, *p* < 0.001]. The body weight was not different between groups, but it was increased over time in animals of all experimental groups (Figure 1A). Analysis of blood glucose levels within and between groups showed no significant effect of treatment [F _(3, 20)_ = 0.0897, *p > *0.05] and time [F _(13, 260)_ = 1.613, *p > *0.05] (Figure 1B).

### 3.2 The effect of intranasal insulin on behavioral performance

**FIGURE 2:**
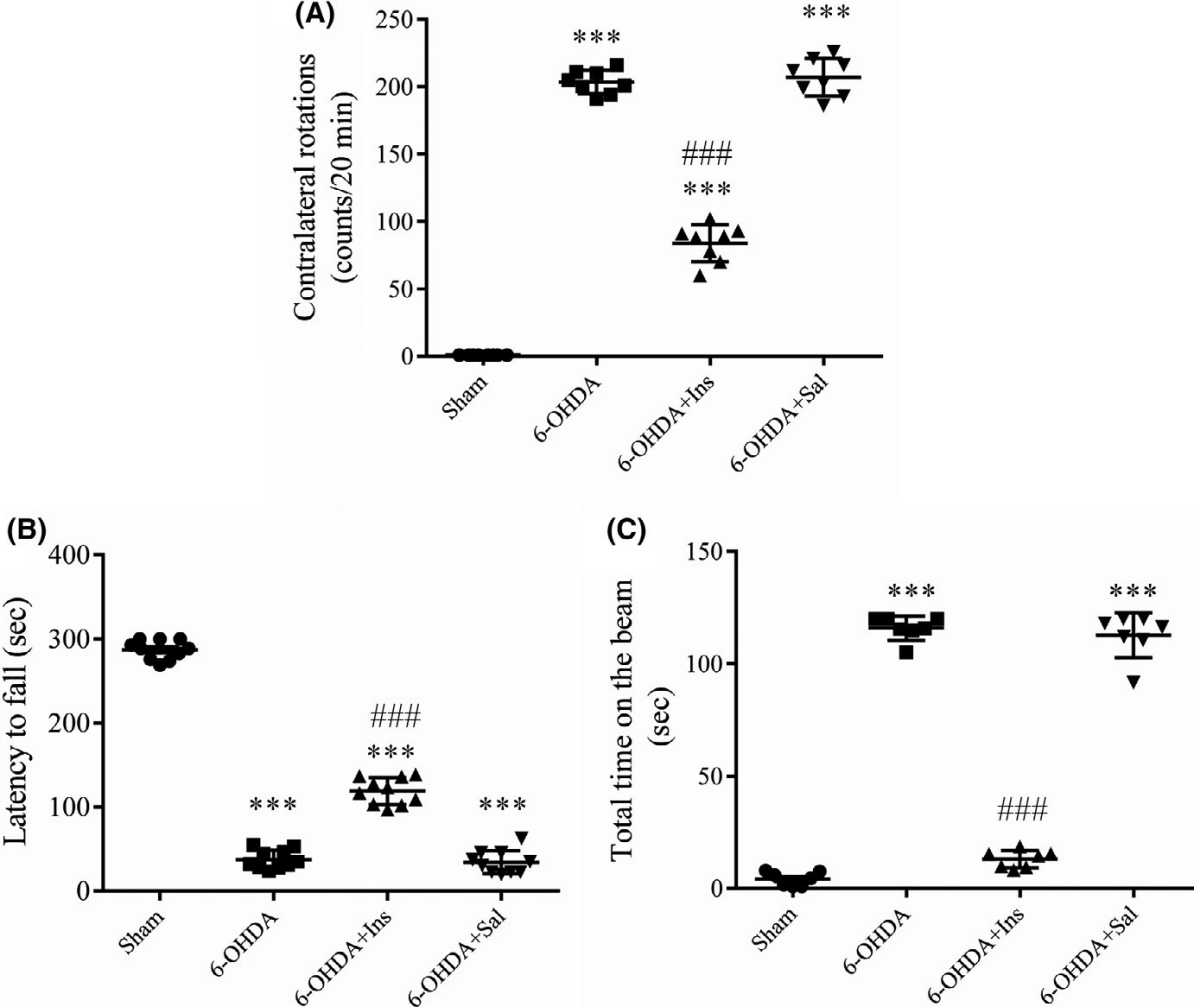
Intranasal insulin administration significantly attenuated motor deficits Motor performance of animals was evaluated using (A) apomorphine‐induced rotation, (B) Rotarod, and (C) Narrow beam tests. Insulin administration could significantly improve motor performance following 6‐OHDA injection. Values are expressed as means ± SEM (*n* = 7–10/group). ^***^ *p* < 0.001 vs. Sham. ^###^ *p* < 0.001 vs. 6‐OHDA

Statistical analysis revealed that there was a significant difference in apomorphine‐induced rotations between groups [F _(3, 28)_ = 697.7, *p* < 0.001]. Contralateral rotations were significantly increased in 6‐OHDA‐induced lesioned rats compared to sham (*p* < 0.001). Insulin administration could decrease (*p* < 0.001) the contralateral rotations exhibited by the untreated lesioned rats but it was yet significantly higher than sham group (*p* < 0.001). No change was observed between 6‐OHDA and 6‐OHDA+sal groups (*p > *0.05) (Figure 2A). Rotarod test was used to evaluate the ability of rats to coordinate the movements on the rotating rod. ANOVA analysis indicated that the latency time to fall was significantly decreased in 6‐OHDA group [F _(3, 36)_ = 810.6, *p* < 0.001] compared to sham. Although there was a significant reduction in the latency time in 6‐OHDA+Ins compared to sham (*p* < 0.001), insulin significantly increased the latency time compared to 6‐OHDA (*p* < 0.001). However, no difference was observed between 6‐OHDA and saline‐treated groups (*p > *0.05) (Figure 2B). Total time on the beam was significantly different between groups [F _(3, 24)_ = 685.4, *p* < 0.001], with significant enhancement in 6‐OHDA group compared to sham (*p* < 0.001). Insulin could reduce it compared to 6‐OHDA (*p* < 0.001), however, no difference was observed between 6‐OHDA and saline‐treated groups (*p > *0.05) (Figure 2C).

### 3.3 Intranasal insulin protected DA neurons of SN against 6‐OHDA

**FIGURE 3:**
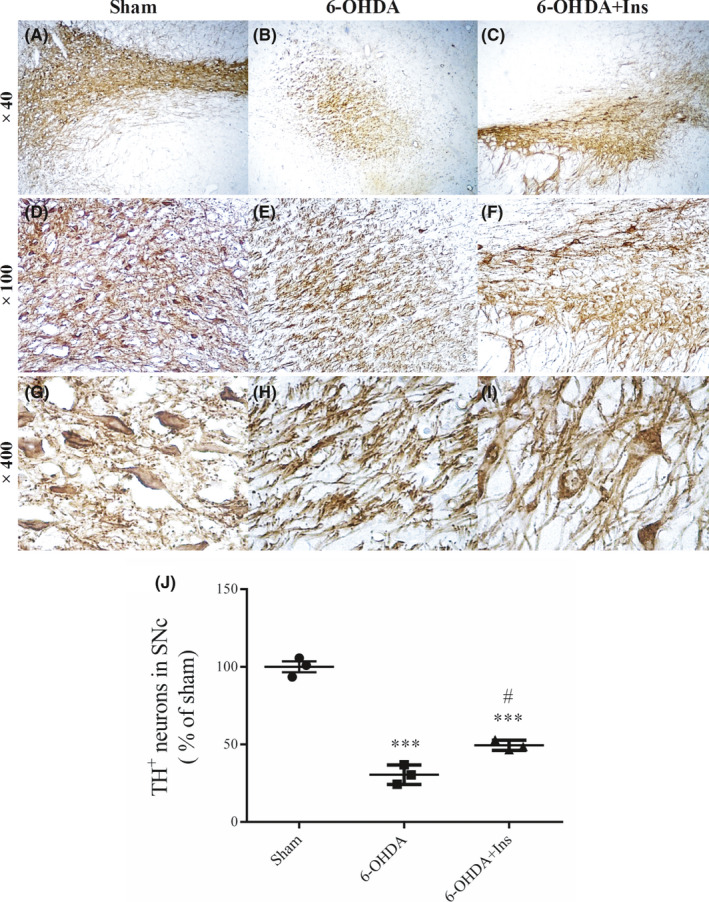
TH‐positive neurons in SNpc. Representative immunostained images of SNpc are shown at (A–C) ×40, (D–F) ×100, and (G–I) ×400 magnifications. (J) The number of TH^+^ dopaminergic neurons in SN was significantly decreased after 6‐OHDA injection, and insulin could increase it. Values are expressed as means ± SEM (*n* = 3/group). ^***^ *p* < 0.001 vs. Sham. ^#^ *p* < 0.05 vs. 6‐OHDA

TH‐positive neurons in the SNpc were evaluated using immunohistochemistry at ×40, ×100 and ×400 magnifications (Figure 3A–I). An obvious reduction in the number of DA neurons was observed in 6‐OHDA group, but insulin administration could significantly prevent neuronal cell death (Figure 3A–I). Data analysis demonstrated that there was a significant loss of DA neurons in the ipsilateral SNpc of 6‐OHDA‐injected rats [F _(2, 6)_ = 132.7, *p* < 0.001]. Although the number of DA neurons was significantly lower in 6‐OHDA+Ins group in comparison to sham (*p* < 0.001), insulin significantly ameliorated 6‐OHDA‐induced DA neurons loss compared to 6‐OHDA group (*p* < 0.05) (Figure 3J). However, there was no significant difference between 6‐OHDA and saline‐treated groups (data not shown).

### 3.4 The effect of intranasal insulin on mitochondrial function indices in striatum after 6‐OHDA injection

**FIGURE 4:**
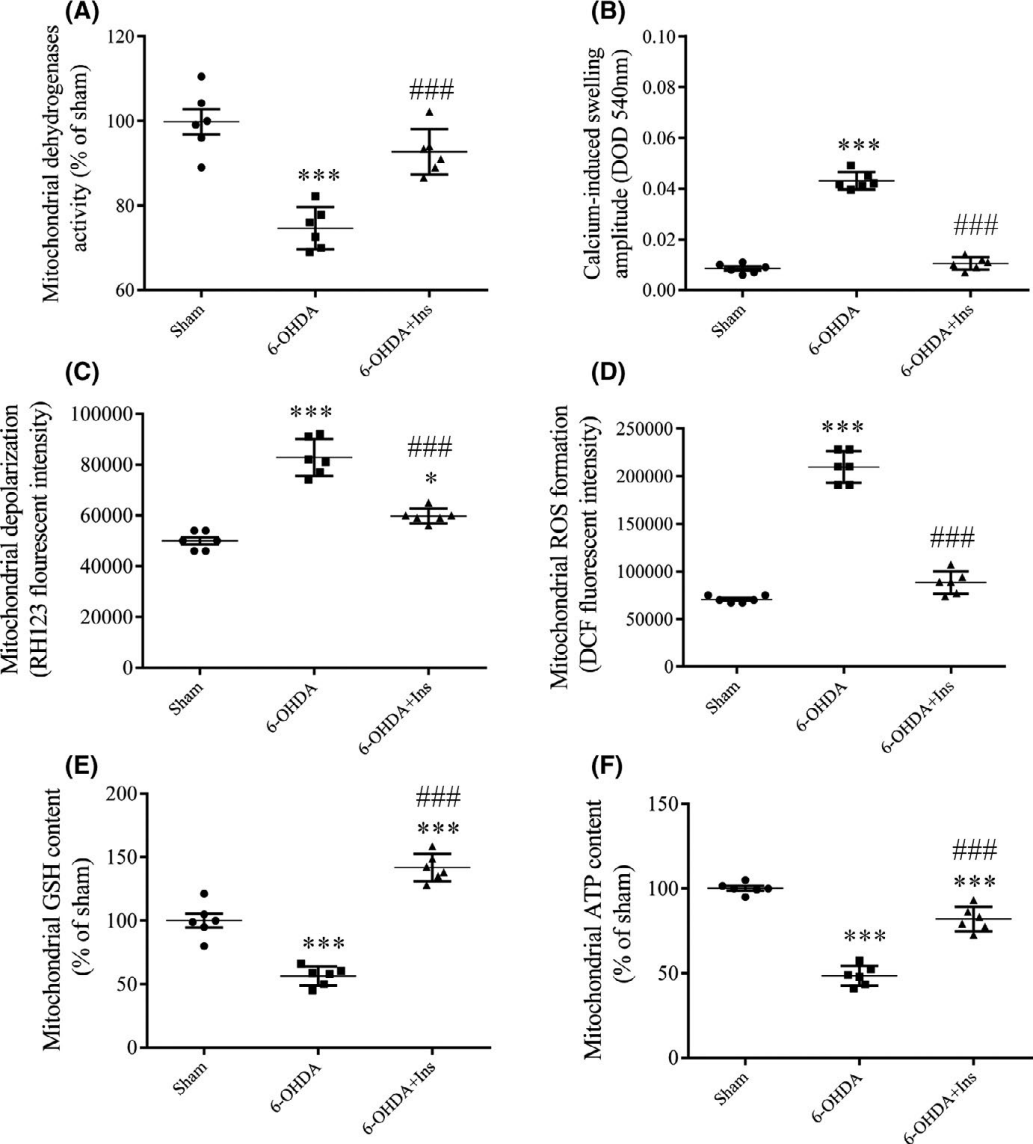
Mitochondrial function indices in striatum. (A) Mitochondrial dehydrogenases activity, (B) mitochondrial swelling, (C) mitochondrial depolarization, (D) mitochondrial ROS formation, (E) glutathione (GSH) content, and (F) mitochondria ATP level were evaluated in mitochondrial extract of striatum. Intranasal insulin administration could significantly attenuate 6‐OHDA‐induced mitochondrial dysfunction. Values are presented as means ± SEM (*n* = 6/group). ^*^ *p* < 0.05, ^***^ *p* < 0.001 vs. Sham. ^###^ *p* < 0.001 vs. 6‐OHDA

Different mitochondrial function indices were evaluated on mitochondria extracted from striatum (Figure 4). ANOVA analysis indicated a significant decrease in mitochondrial dehydrogenases activity [F _(2, 15)_ = 28.24, *p* < 0.001] and a significant increase in mitochondrial swelling [F _(2, 15)_ = 319.7, *p* < 0.001] in 6‐OHDA group compared to sham. Insulin treatment could significantly increase dehydrogenases activity and decrease mitochondrial swelling compared to 6‐OHDA (*p* < 0.001 and *p* < 0.001, respectively) (Figure 4A,B). Mitochondrial depolarization and ROS formation were significantly increased in 6‐OHDA group compared to sham ([F _(2, 15)_ = 68.39, *p* < 0.001] and [F _(2, 15)_ = 239.7, *p* < 0.001], respectively). Although mitochondrial depolarization was significantly higher in 6‐OHDA+Ins than sham (*p* < 0.05), insulin could decrease it compared to 6‐OHDA (*p* < 0.001) (Figure 4C). ROS formation was also significantly reduced in 6‐OHDA+Ins group in comparison to 6‐OHDA (*p* < 0.001) (Figure 4D). Statistical analysis indicated that GSH and ATP levels in striatum were decreased in 6‐OHDA‐injected group ([F _(2, 15)_ = 92.79, *p* < 0.001] and [F _(2, 15)_ = 126.3, *p* < 0.001], respectively). Insulin treatment significantly enhanced GSH level in 6‐OHDA+Ins group in comparison to both sham (*p* < 0.001) and 6‐OHDA (*p* < 0.001) (Figure 4E). Although, a significant reduction in ATP level was observed in 6‐OHDA+Ins compared to sham (*p* < 0.001), insulin could increase it in comparison to 6‐OHDA (*p* < 0.001) (Figure 4F).

### 3.5 The effects of insulin on the expression of genes involved in mitochondrial biogenesis and fission in striatum

**FIGURE 5:**
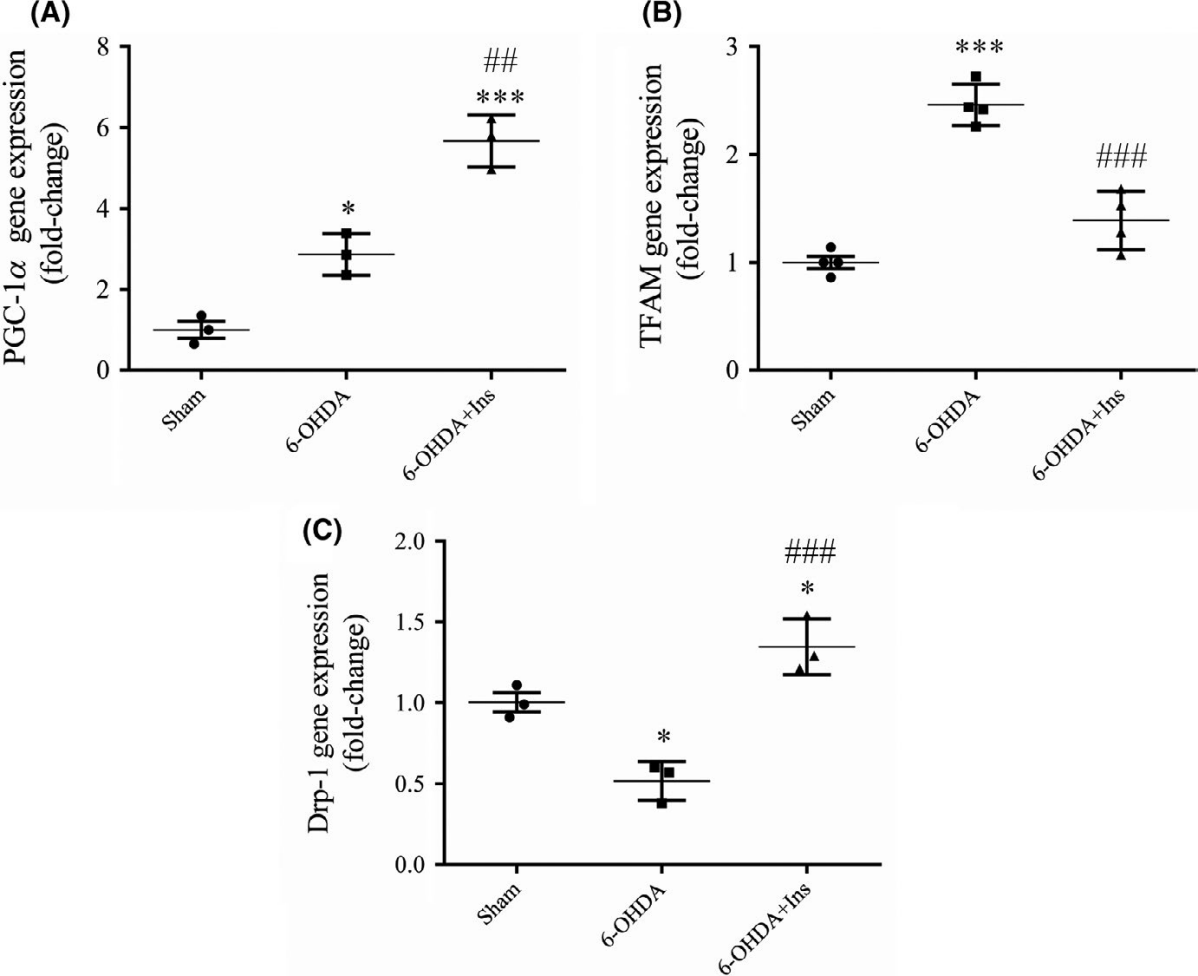
The expression of genes involved in mitochondrial biogenesis and fission. The expression of (A) PGC‐1α and (B) TFAM was increased after 6‐OHDA injection. Insulin treatment could increase PGC‐1α and decrease TFAM genes expression. (C) The expression of Drp‐1 was reduced in 6‐OHDA group and enhanced by insulin administration. Data are expressed as means ± SEM (*n* = 3–4/group). ^*^ *p* < 0.05, ^***^ *p* < 0.001 vs. Sham. ^##^ *p* < 0.01 and ^###^ *p* < 0.001vs. 6‐OHDA

A significant change was observed in the expression of PGC‐1α [F _(2, 6)_ = 62.24, *p* < 0.001], TFAM [F _(2, 9)_ = 56.05, *p* < 0.001] and Drp‐1 [F _(2, 6)_ = 28.99, *p* < 0.001] between groups (Figure 5). An increased expression of PGC‐1α was observed in 6‐OHDA and 6‐OHDA+Ins groups compared to sham (*p* < 0.05 and *p* < 0.001, respectively). However, it was significantly higher in 6‐OHDA+Ins group in comparison to 6‐OHDA (*p* < 0.01) (Figure 5A). ANOVA analysis indicated that 6‐OHDA increased TFAM gene expression compared to sham (*p* < 0.001), and insulin could significantly attenuate it (*p* < 0.001) in comparison to 6‐OHDA (Figure 5B). Tukey’s post hoc test indicated that although 6‐OHDA decreased Drp‐1 gene expression compared to sham (*p* < 0.05) insulin significantly enhanced it compared to both sham and 6‐OHDA groups (*p* < 0.05 and *p* < 0.001, respectively) (Figure 5C).

### 3.6 Insulin treatment elevated GFAP and Iba‐1 mRNA levels in striatum

**FIGURE 6:**
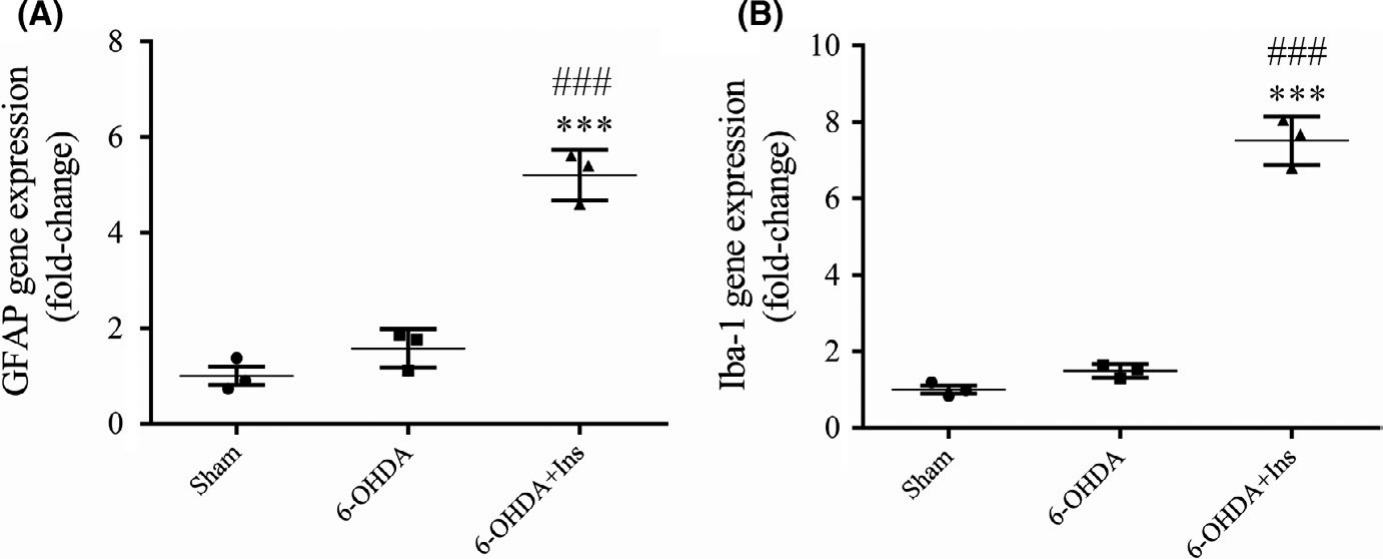
The expression of genes involved in astrocyte and microglia activity in striatum. Although 6‐OHDA had no effect on (A) GFAP and (B) Iba‐1 mRNA levels, as astrocyte and microglia activity markers, insulin significantly increased them. Data are expressed as means ± SEM (*n* = 3/group). ^***^ *p* < 0.001 vs. Sham. ^###^ *p* < 0.001 vs. 6‐OHDA

One‐way ANOVA demonstrated a significant difference in the expression of GFAP and Iba‐1 genes between group ([F _(2, 6)_ = 83.96, *p* < 0.001] and [F _(2, 6)_ = 249.6, *p* < 0.001], respectively) (Figure 6). Although 6‐OHDA had no effect on the mRNA levels of GFAP and Iba‐1 (*p > *0.05 compared to sham), insulin administration significantly increased them in comparison to both sham (*p* < 0.001) and 6‐OHDA (*p* < 0.001) (Figure 6A,B).

## 4 DISCUSSION

In current study, we found that IN insulin could rescue dopaminergic neurons against cell death induced by 6‐OHDA, and ameliorate motor deficits in a rat model of PD, which is consistent with previous studies.^9^, ^11^

6‐OHDA undergoes oxidation inside the cells, produces reactive oxygen species (ROS), and results in mitochondrial enzymes dysfunction, mtDNA mutation, disruption of mitochondrial membrane permeability and apoptotic cell death.^29^, ^30^ Also, excessive free radicals induced by 6‐OHDA may open the mPTP,^31^ and drive some antioxidant molecules such as GSH to exit from mitochondria. Therefore, ability of the mitochondria will not be enough to neutralize ROS, which in turn results in more ROS production. Furthermore, induction of mPTP increases mitochondrial membrane permeability and causes mitochondria to become more depolarized, therefore, Δψ_m_ will be abolished resulting outflow of protons across the outer mitochondrial membrane and eventually disruption in ATP generation.^14^ Similarly, we observed increased ROS formation and mitochondrial membrane permeability, decreased GSH content and ATP level in striatum of 6‐OHDA‐lesioned rats. Interestingly, we observed that treatment with insulin attenuated all detrimental effects of 6‐OHDA. Previous studies have also found that insulin decreases ROS production in the neurons^32^, ^33^ and it has an important role in enhancement of Δψ_m_, intracellular ATP levels and NADPH redox state, through activation of PI3K/Akt/CREB pathway, and therefore, it can improve axonal outgrowth.^34^

Producing the new mitochondria is controlled by mitochondrial biogenesis pathway.^35^ In the context of neural damage, mitochondrial biogenesis can be activated as a compensatory mechanism to protect the neurons. It has been reported that excessive production of ROS increases PGC‐1α in neurons after ischemia, which triggers upregulation of mitochondrial ROS‐detoxifying enzymes such as glutathione peroxidase (GPx), catalase, and superoxide dismutase (SOD).^36^ It has been also declared that IN insulin enhances PGC‐1α protein level and ATP production, supporting the direct role of insulin in promoting mitochondrial biogenesis and ATP production in the brain.^37^ PI3K/Akt activation is associated with activation of PGC‐1α.^38^ In our study, both 6‐OHDA and insulin increased PGC‐1α expression, however, increment of the expression level by insulin was much more than 6‐OHDA. PGC‐1α can stimulate the expression of NRF1 and coactivate the transcription function of NRF1 on the promoter of TFAM.^39^ Surprisingly, our data showed that despite of increasing in TFAM expression in 6‐OHDA‐lesioned rats, insulin treatment had no effect on it. This observation may be explained by reports that suggest the molecular pathways involved in TFAM expression are different between human and rodents. NRF1 and 2 are two trans‐acting factors that play an important role in the transcription of the human TFAM gene.^40^ But NRF1 sites do not appear to be conserved in the TFAM promoters in rodents, while NRF2 sites are present in both humans and rodents.^41^ It has been revealed that oxidative stress leads to translocation of NRF2 into the nucleus and bond with antioxidant response element (ARE), activating a large group of genes associated with the antioxidant and detoxifying response^42^ and increase TFAM expression. Therefore, it can be concluded that in rodents, NRF2 is responsible for TFAM activation rather than NRF‐1 so that increased oxidative stress induced by 6‐OHDA causes an increase in NRF‐2 which eventually leads to enhancement of TFAM gene expression and deal with inflammation.

Another important phenomena involved in adaptation of mitochondrial function in various conditions is dynamic structural changes in the mitochondrial network, including continuous mitochondrial remodeling by fusion and fission processes.^14^, ^16^ In mammals, central player of fission is a GTPases dynamin‐related protein (Drp1).^14^ In current study, Drp‐1 gene expression was partially decreased in 6‐OHDA‐lesion rats and insulin could increase it. It has previously showed that nigrostriatal DA neurons require Drp‐1 to maintain their axons to survive and DA neurons lacking Drp‐1 have less mitochondrial mass in their cell body and axons,^43^ and consequently, have lower ATP levels.^44^ The underlying mechanisms of mitochondrial depletion in axons are the lower overall mitochondrial mass in the cell, and slow mitochondrial movement through the axon due to the large size of them.^43^ DA neurons of SNpc have unusually high energy demands due to their unmyelinated structure and having the large projection fields,^13^ so, they are particularly dependent on the mitochondrial mass and distribution in their processes. However, some studies have been represented that an unusual increase in Drp‐1 expression reduces mitochondrial mass due to enhanced mitochondrial fission and subsequent removing the fragmented mitochondria via mitophagy.^45^, ^46^

It has been indicated that astrocytes may release extracellular mitochondrial particles that could enter into neurons to improve the viability of the cells, and therefore, increase the recovery after stroke.^47^ In addition, astrocytes are essential components of antioxidant defense system in the brain which regulate extracellular concentrations of glutamate and antioxidant compounds.^48^ Therefore, increase in astrocyte function may also contribute to decrease oxidative damage in PD which has been previously reported.^49^ Astrocytes are also an integral component of the antioxidant defense system in the brain through the regulation of extracellular glutamate concentrations and production of antioxidant compounds.^48^, ^49^, ^50^. Recently, it has been identified two types of reactive astrocytes, neurodegenerative A1 types and neuroprotective A2 types.^51^ A1 types, release neurotoxins that lead to cell death and development of neurodegenerative diseases, while A2 type, support neuronal survival and tissue repair through expression and releasing various trophic factors such as transforming growth factor‐β (TGF‐β) which plays a neuroprotective role and participates in synaptogenesis.^52^, ^53^ It has been revealed that reduced PI3K/Akt signaling activation leads to increase in the A1 phenotype, while increased activation of the PI3K/Akt pathway was accompanied by transformation to the A2 phenotype.^53^, ^54^ On the other hand, Insulin may cause GFAP augmentation and astroglial hypertrophy through PI3K/Akt‐dependent pathway.^55^ Furthermore, pretreatment with the rosiglitazone (an insulin sensitizer) attenuates striatal DA neurodegeneration in 6‐OHDA‐lesioned model of PD by increase in GFAP expression and astrocyte function in the SNpc and striatum.^56^ The number of GFAP‐positive cells has been shown to inversely correlates with DA cells loss.^57^ Consistent with these findings, we observed that insulin treatment increased GFAP gene expression in the striatum of rats indicating that the neuroprotective effects of insulin are mediated, at least in part, by modulation of A1/A2 astrocytic conversion through upregulation of the PI3K‐Akt pathways.

Microglial activation was also observed after insulin treatment, characterized by increased Iba‐1 gene expression in striatum. It has been proposed two subpopulation of active microglia, M1 phenotype and M2 phenotype.^58^ M1 microglia activation leads to production of several pro‐inflammatory cytokines such as ROS and tumor necrosis factor‐a (TNF‐α). Conversely, M2 microglia activation alleviates inflammatory response and promotes tissue repair by releasing the anti‐inflammatory cytokines include interleukin‐4 (IL‐4), IL‐10 and various trophic factors such as TGF‐β.^59^ Recently, some increasing evidence has revealed that PI3K/Akt pathway plays an important role in microglial M1/M2 polarization via induction of M2‐type cell accumulation and the inhibition of M1‐type microglia production.^60^, ^61^ A study also showed that insulin treatment reduces the release of pro‐inflammatory cytokine TNF‐α^62^ and induces an increase in the TGF‐β receptors at the cell surface which causes enhancement in the cell responsiveness to autocrine TGF‐β.^63^ Although the molecular mechanisms of TGF‐β to promote anti‐inflammatory effects of microglia are less understood, an in vitro study has been shown that TGF‐β1 (an isoform of TGF‐β) might regulate different states of microglia activation by inhibition of M1 form, and shifting toward M2 phenotypes.^64^ Therefore, insulin likely upregulates anti‐inflammatory phenotypes of reactive glia via PI3K/Akt pathway which it may be at least a part of neuroprotective effects of insulin.

## 5 CONCLUSION

The findings of the present study demonstrated that IN administration of insulin attenuates the motor impairments following 6‐OHDA injection in MFB as an experimental model of PD. The protective effects of insulin are mediated, at least in part, by improvement in mitochondrial function indices, mitochondrial biogenesis and fission as well as activation of anti‐inflammatory/neuroprotective phenotype of astrocytes and microglia. These findings suggest that IN insulin can be considered as a promising treatment for patients with PD.

## CONFLICTS OF INTEREST

The authors declare that they have no conflict of interest.

## AUTHOR CONTRIBUTION

Conception and design of the study: Abolhassan Ahmadiani, Farideh Iravanpour, Leila Dargahi, Mohsen Rezaei, Reza Heidari. Performing the research: Farideh Iravanpour. Analysis and interpretation of data: Abolhassan Ahmadiani, Farideh Iravanpour, Leila Dargahi, Masoud Haghani, Neda Valian. Writing the article: Farideh Iravanpour. Revising the article critically for important intellectual content: Abolhassan Ahmadiani, Leila Dargahi, Neda Valian, Mohsen Rezaei, Masoud Haghani, Reza Heidari.

## ETHICAL APPROVAL

All the experiments were performed in conformity with the guidelines for care and use of experimental animals which were approved by the ethics Committee of Shahid Beheshti University of Medical Sciences, Tehran, Iran (IR.SBMU.MSP.REC.1397.54).

## Data Availability

The datasets generated and analyzed during the current study are available from the corresponding author on reasonable requests.
